# Sex differences in tuberculosis incidence: a pooled analysis of 11 prospective cohort studies

**DOI:** 10.1016/j.eclinm.2026.104146

**Published:** 2026-08-12

**Authors:** Yohhei Hamada, Sujen Surenthirarajah, Rishi K. Gupta, Matteo Quartagno, Carlos Acuna-Villaorduna, Neus Altet, Roland Diel, Jose Dominguez, Sian Floyd, Amita Gupta, Edward C. Jones-López, Christoph Lange, Frank van Leth, Irene Latorre Rueda, Vidya Mave, Laura Muñoz, Mandar Paradkar, Martina Sester, Haridas Prasad, Kwame Shanaube, Surendra K. Sharma, Rosa Sloot, Giovanni Sotgiu, Richa Vashishtha, Ibrahim Abubakar, Molebogeng X. Rangaka

**Affiliations:** a Institute for Global Health, University College London, London, United Kingdom; b MRC Clinical Trials Unit, Institute of Clinical Trials and Methodology, University College London, London, United Kingdom; c Boston University Medical Center, Section of Infectious Diseases, Boston, MA, USA; d Unitat de Tuberculosis, Hospital Universitari Vall d’Hebron-Drassanes, Unitat de TDO de la Tuberculosis ‘Servicios Clínicos’, Barcelona, Spain; e Institute for Epidemiology, University Hospital Schleswig-Holstein, Campus Kiel, Kiel, Germany; f Institut d'Investigació Germans Trias i Pujol, CIBER Enfermedades Respiratorias, Instituto de Salud Carlos III, Universitat Autònoma de Barcelona, Barcelona, Spain; g Department of Infectious Disease Epidemiology, London School of Hygiene & Tropical Medicine, London, United Kingdom; h Johns Hopkins University School of Medicine, Baltimore, MD, USA; i Division of Infectious Diseases, Department of Medicine, Keck School of Medicine of USC, University of Southern California, CA, USA; j Division of Clinical Infectious Diseases, Research Center Borstel, Borstel, Germany; k German Center for Infection Research (DZIF), Clinical Tuberculosis Unit, Borstel, Germany; l Respiratory Medicine & International Health, University of Lübeck, Germany; m Baylor College of Medicine and Texas Children's Hospital, Houston, TX, USA; n Department of Health Sciences, VU University, Amsterdam Public Health Research Institute, Amsterdam, the Netherlands; o Byramjee Jeejeebhoy Government Medical College-Johns Hopkins University Clinical Research Site, Pune, Maharashtra, India; p Center for Infectious Diseases in India, Johns Hopkins India, Pune, Maharashtra, India; q Department of Clinical Sciences, University of Barcelona, Barcelona, Spain; r Parc Sanitari Sant Joan de Déu (Sant Boi), Barcelona, Spain; s Department of Transplant and Infection Immunology, Saarland University, Homburg, Germany; t Center for Gender-specific Biology and Medicine (CGBM), Saarland University, Homburg, Germany; u Byramjee Jeejeebhoy Government Medical College and Sassoon General Hospital, Pune, Maharashtra, India; v Zambart, Lusaka, Zambia; w Department of Internal Medicine, All India Institute of Medical Sciences, New Delhi, India; x Department of Molecular Medicine, Jamia Hamdard Institute of Molecular Medicine, Hamdard University, Delhi, India; y Departments of General Medicine & Pulmonary Medicine, JNMC, Datta Meghe Institute of Medical Sciences, Wardha, Maharashtra, India; z Desmond Tutu TB Centre, Department of Paediatrics and Child Health, Faculty of Medicine and Health Sciences, Stellenbosch University, Cape Town, South Africa; aa Clinical Epidemiology and Medical Statistics Unit Department of Medicine, Surgery and Pharmacy, University of Sassari, Sassari, Italy; ab Faculty of Population Health Sciences, University College London, London, United Kingdom; ac School of Public Health, and Clinical Infectious Disease Research Institute-AFRICA, University of Cape Town, South Africa

**Keywords:** Tuberculosis, Sex factors, Disease progression, Latent tuberculosis, Epidemiologic factors, Prospective cohort studies

## Abstract

**Background:**

Tuberculosis (TB) incidence is consistently higher in men than women globally. Whether this disparity arises from differential infection risk or sex-specific rates of progression from infection to disease remains unclear. We evaluated sex differences in the development of TB disease while accounting for baseline infection status.

**Methods:**

We conducted a pooled individual participant data analysis of 11 prospective cohort studies (n = 22,424), conducted between 2005 and 2017 across Sub-Saharan Africa, Europe, Asia, and South America. TB infection was defined by a positive QuantiFERON-TB Gold In-Tube result, and incident TB was defined by bacteriologically confirmed or clinical diagnosis. We used mixed-effects logistic and Cox proportional hazards models, adjusted for age, to estimate odds ratios (OR) for infection and hazard ratios (HR) for incident TB by sex.

**Findings:**

Participants were followed for a median of 2.8 years, during which 411 individuals (1.8%) developed TB. Most participants were from Europe (52%) or South Africa/Zambia (34.4%). At baseline, males had a significantly higher likelihood of infection (OR 1.12; 95% CI 1.02–1.22). Among those with baseline infection, pooled incidence rates were 16.3 per 1000 person-years (95% CI 10.9–24.4) in females and 15.6 (95% CI 9.8–24.8) in males. The risk of developing TB did not differ by sex among those with baseline infection (HR 0.86; 95% CI 0.65–1.14) or without baseline infection (HR 1.02; 95% CI 0.71–1.47).

**Interpretation:**

In these cohorts, males had a higher prevalence of baseline TB infection, but no clear excess risk of subsequent TB disease after accounting for baseline infection status. These findings suggest that sex differences in TB infection risk may contribute more to higher TB incidence among males than differences in TB disease risk after infection.

**Funding:**

None.

**Research in context**

**Evidence before this study**

Programmatic notification data consistently show a higher global burden of tuberculosis (TB) in men compared to women. The exact mechanisms driving this disparity have remained unclear. Recent systematic reviews have reported a higher prevalence of TB infection in men, suggesting that the increased risk of active TB may be primarily driven by differential exposure. We searched MEDLINE from inception to January 23, 2026, using terms related to “tuberculosis,” “sex,” and “cohort studies”. This search identified limited research that longitudinally evaluated TB risk by sex while accounting for baseline TB infection status. An early study from India (ICMR 2003) reported a higher risk of TB in males over 25 years of follow-up among both tuberculin skin test (TST) positive and negative individuals. A more recent study (Chaisson 2025) in Brazil reported higher TB incidence in men living with human immunodeficiency virus compared to women, despite both sexes having similar TST reactivity at baseline. However, we found no contemporary studies evaluating TB risk across diverse settings and populations using prospective cohorts with baseline interferon-gamma release assay results.

**Added value of this study**

We collated data from 11 prospective cohorts and analysed the risk of TB infection and incident TB development accounting for baseline QuantiFERON-TB Gold In-Tube results. The analysis found a higher risk of TB infection in men but did not find a significant difference in the risk of TB incidence by sex among those with baseline infection or without baseline infection.

**Implications of all the available evidence**

Our results suggest that differences in exposure and infection risk may be important contributors to the higher global incidence of TB in men, rather than a large difference in disease progression after infection. This underscores the importance of tailoring TB prevention and screening strategies to address sex-specific exposure risk.

## Introduction

Tuberculosis (TB) remains a major global health challenge. The World Health Organization (WHO) estimates that approximately 10.7 million people developed TB in 2024, with incidence consistently higher in males than in females.^1^ This disparity is pronounced especially in adults. WHO estimates 5.8 million incident adult males compared with 3.7 million adult females, which corresponds to a 1.6-fold higher incidence in males.^1^ The precise mechanisms behind this difference, whether related to biological susceptibility, differential exposure, or variation in progression from infection to disease, are not yet fully understood.

The higher burden of TB among males is well documented: a meta-analysis of TB prevalence surveys found a two-fold higher prevalence in males, with differences in care-seeking or reporting unable to fully explain the observed gap.^2^ Proposed factors include greater social mixing, longer duration of infectiousness, and preferential mixing among males.^3^^,^^4^ No sex differences in TB infection among children, but adult males show a steadily increasing risk that reaches about 1.3-fold higher prevalence by age 40,^4^ indicating that exposure dynamics may be a key determinant of sex disparities. Furthermore, biological sex-related differences could be attributed to innate and adaptive immune responses.^5^

Despite this evidence, it is difficult to distinguish the contributions of differential infection risk from differences in progression to disease in explaining higher TB incidence in males. Prevalence surveys can help address differences in care-seeking and reporting, but they cannot determine whether the higher male burden reflects a higher risk of acquiring TB infection, a higher risk of progression after infection, or both. To address this gap, we conducted a pooled individual participant data analysis from multiple cohort studies that prospectively followed individuals who had undergone baseline TB infection testing. This approach allowed us to evaluate sex differences in the development of TB while accounting for baseline infection status.

## Methods

### Study design

Anonymised individual participant data from prospective cohort studies included in our previously published systematic review and meta-analysis were used.^6^ Briefly, the dataset was originally assembled to compare the predictive value of the tuberculin skin test (TST) and interferon-gamma release assays (IGRAs) for incident TB. We conducted a systematic literature search to identify studies that systematically tested participants with both TST and IGRA and prospectively followed them for incident TB for a median duration of at least one year in any geographical setting.

The original review included 13 studies, of which 11 were included in the present analysis.^7^, ^8^, ^9^, ^10^, ^11^, ^12^, ^13^, ^14^, ^15^, ^16^, ^17^ Two were not included: one reported no incident TB during follow-up,^18^ and for the other, permission to use the dataset was not granted by the Authors.^19^ The 11 included studies, comprising 27,538 participants, were conducted between 2005 and 2017 across a wide range of settings, including Sub-Saharan Africa (South Africa and Zambia), Europe (Spain, Germany, the UK, Bulgaria, Portugal, Italy, the Netherlands, Romania, Greece, Switzerland, Denmark, and Turkey), India, and Brazil.

### Ethics statement

Ethical approvals for sharing anonymised individual participant data were obtained by data contributors, where required. Each original study obtained informed consent from participants. The present study was approved by the University College London Research Ethics Committee (ref: 1013) and was conducted in accordance with the principles of the Declaration of Helsinki.

### Definitions

Incident TB was defined as bacteriologically confirmed or clinically diagnosed TB, as per each study's definitions. In the sensitivity analysis, we restricted to bacteriologically confirmed TB. We excluded cases of prevalent TB defined as TB disease diagnosed within 42 days of enrolment.^6^

TB infection was defined as a positive QuantiFERON-TB Gold In-Tube (QFT) result as per its package insert without TB.^20^ QFT was prioritized over T-SPOT. TB or TST due to its consistent availability across all datasets and its superior specificity to the TST.

The original studies variably described the male/female variable as sex or gender. However, there was insufficient information to determine whether this variable was deliberately collected as biological sex, self-reported gender, or administrative sex in each cohort. We therefore refer to this harmonised binary male/female variable as recorded sex throughout the manuscript, while conceptualising observed differences as potentially reflecting both biological sex-related mechanisms and socially patterned gender-related factors (Figure S1).

### Statistical analysis

We excluded participants with missing data on sex, missing or indeterminate QFT results, prevalent TB at baseline, and those who received TB preventive treatment.

For each study, TB infection prevalence was estimated by sex and pooled estimates were calculated across studies using random-effects meta-analysis implemented with a generalized linear mixed model. The Hartung–Knapp (HK) adjustment was applied to derive confidence intervals (CI) for the pooled random-effects estimates.^21^ To estimate the association between sex and TB infection, we conducted a one-stage meta-analysis using mixed-effects logistic regression models. Study was included as a fixed effect to allow for study-specific differences in baseline TB infection prevalence, and the sex effect was allowed to vary across studies through a random slope for sex.

Second, we calculated TB incidence stratified by sex and baseline TB infection status and pooled these estimates across studies using a generalized linear mixed model with the HK adjustment. To estimate the association between sex and subsequent TB disease, we used mixed-effects Cox proportional hazards models stratified by baseline TB infection status. These models similarly included study as a fixed effect and a random slope for sex by study, allowing the association between sex and incident TB to vary across cohorts.

The primary aim of these analyses was to estimate the overall association between recorded sex and TB infection or subsequent TB disease risk, rather than to isolate a direct biological effect of sex independent of gender-related behavioural, social, healthcare, or immunological pathways (Figure S1). Therefore, models were adjusted for age only (modelled as a continuous linear term), because age is a key determinant of TB infection and progression to disease and may differ between males and females. We did not adjust for additional covariates such as smoking status, country-of-birth TB incidence category, or reason for screening because these variables may partly mediate the association between recorded sex and TB infection or disease, or reflect study population selection. Additional adjustment could therefore block part of the overall association between recorded sex and TB outcomes. Between-study differences were accounted for using the mixed-effects structure described above.

We assessed the proportional hazards assumption for each model using Schoenfeld residuals in Cox models stratified by study.^22^

To assess heterogeneity, all pooled estimates were presented using forest plots and I-squared statistics were estimated. In addition, subgroup analyses were conducted by human immunodeficiency virus (HIV) status, TB contact status (history of contact with a person with TB vs. none), national TB incidence in study countries (<100 vs. ≥100 per 100,000 population at the time of the study), and age (≥18 vs. <18 years). Within each subgroup, TB incidence rates were estimated by sex and fitted age-adjusted Cox models.

Sensitivity analyses were conducted for the primary analysis (the age-adjusted hazard ratio for incident TB). These included the age-adjusted hazard ratio using a two-stage random-effects meta-analysis, fitting a Poisson regression model, and restricting the outcome definition to bacteriologically confirmed TB.

All analyses were conducted using R statistical software, version 4.3.1.

### Role of the funding source

There was no funding source for this study.

## Results

**Fig. 1:**
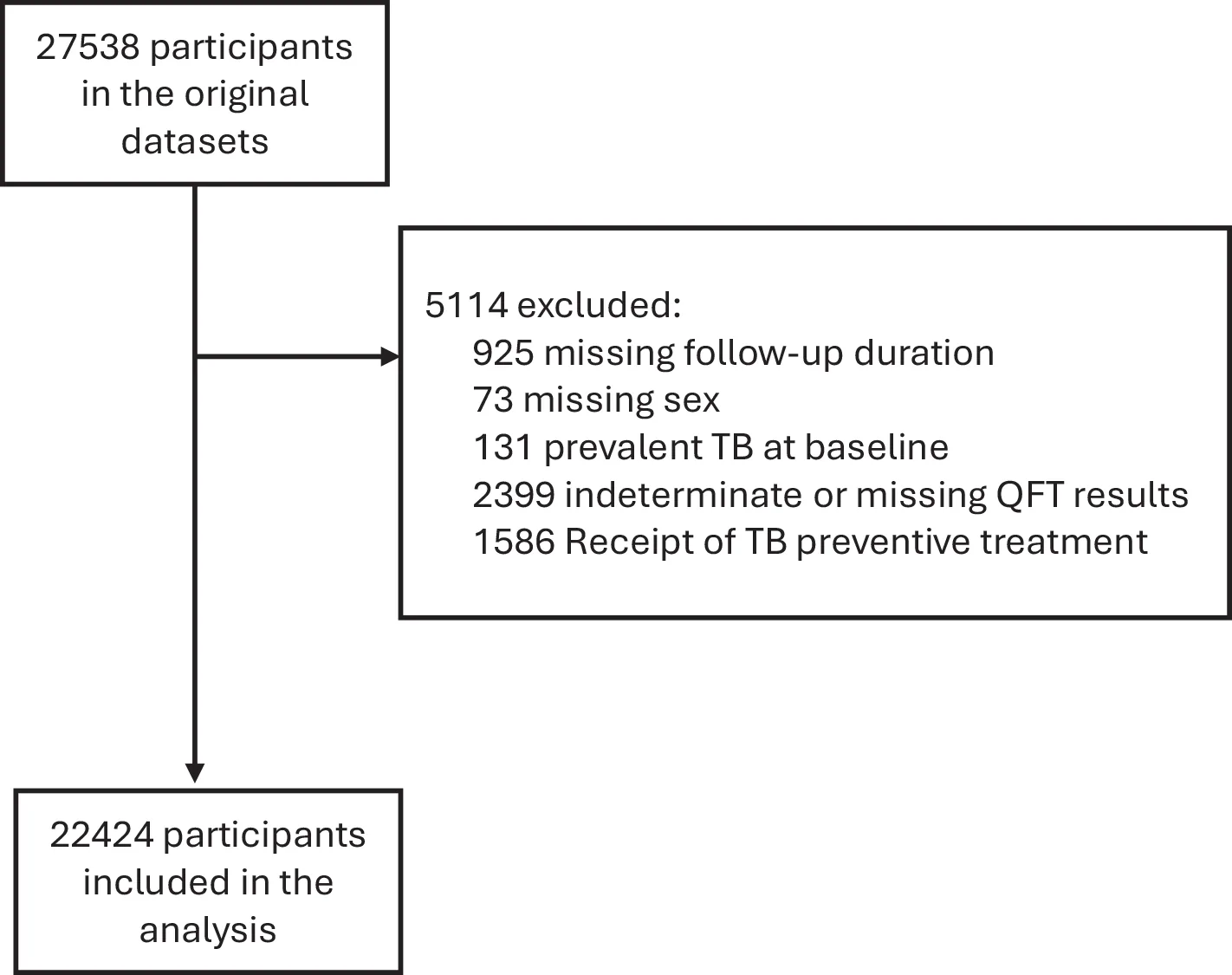
**Flow diagram**. TB: tuberculosis: QFT: QuantiFERON.

Of the 27,538 participants from 11 included studies, 5114 were excluded because of missing data, prevalent TB at baseline, indeterminate QFT results, or receipt of TB preventive treatment (Fig. 1). Among 1586 participants who were excluded because they received TB preventive treatment, 888 (56.0%) were male and the mean age was 26.8 years (Standard deviation: 14.7). This group included 530 (33.4%) people living with HIV, of whom 458 were enrolled in a randomized trial of tuberculosis preventive treatment in South Africa.^15^ After exclusions, 22,424 participants were included in the analysis.

Participants were followed for a median (interquartile range, IQR) of 2.8 (2.0–4.5) years, during which 411 (1.8%) developed TB. Most participants were from European countries (n = 11,661; 52%), followed by South Africa or Zambia (n = 7716; 34.4%), India (n = 2418; 10.8%), and Brazil (n = 629; 2.8%) (Table 1). The most common reason for screening was contact investigation (46%). Overall, 24% (n = 5457) were born in countries with a TB incidence rate <100 per 100,000 population, and 6.6% (n = 1491) were living with HIV. Adults aged ≥18 years comprised the majority of the study population (n = 14,979, 66.8%). Adolescents aged 12–17 years accounted for 30.0% (n = 6727), largely reflecting one South African study that exclusively enrolled adolescents in this age group^12^; children aged <12 years accounted for 3.3% (n = 740). Table S1 presents the participant characteristics by study.

**Table 1:** Characteristics of participants.

	Level	Overall	Female	Male
n		22,424	11,735	10,689
Age (mean (SD))		29.69 (16.52)	29.72 (16.62)	29.66 (16.40)
HIV status (%)	Negative	18,929 (84.4)	9951 (84.8)	8978 (84.0)
	Positive	1491 (6.6)	818 (7.0)	673 (6.3)
	Missing	2004 (8.9)	966 (8.2)	1038 (9.7)
History of contact with a person with TB (%)	No	10,525 (46.9)	5355 (45.6)	5170 (48.4)
	Yes	11,891 (53.0)	6374 (54.3)	5517 (51.6)
	Missing	8 (0.0)	6 (0.1)	2 (0.0)
Smoking status (%)	Ever smoker	2630 (11.7)	755 (6.4)	1875 (17.5)
	Never smoker	11,280 (50.3)	6419 (54.7)	4861 (45.5)
	Missing	8514 (38.0)	4561 (38.9)	3953 (37.0)
Body mass index (mean (SD))		24.08 (5.51)	24.28 (5.82)	23.84 (5.12)
	Missing (%)	7273 (32.4)	3629 (30.9)	3644 (34.1)
TB incidence rate (per 100 K) in country of birth	<100	5457 (24.3)	2693 (22.9)	2764 (25.9)
	100–300	7770 (34.7)	3798 (32.4)	3972 (37.2)
	>300	8158 (36.4)	4765 (40.6)	3393 (31.7)
	Missing	1039 (4.6)	479 (4.1)	560 (5.2)
Reason for TB infection screening (%)	Contact investigation	10,311 (46.0)	5481 (46.7)	4830 (45.2)
	Migrant screening	4207 (18.8)	2080 (17.7)	2127 (19.9)
	HIV	1092 (4.9)	523 (4.5)	569 (5.3)
	Immunosuppression (other than HIV)	695 (3.1)	335 (2.9)	360 (3.4)
	Research purpose (HIV-negative adolescents)	6037 (26.9)	3272 (27.9)	2765 (25.9)
	Missing	82 (0.4)	44 (0.4)	38 (0.4)
Previous TB diagnosis (%)	No	16,964 (75.7)	8744 (74.5)	8220 (76.9)
	Yes	1231 (5.5)	663 (5.6)	568 (5.3)
	Missing	4229 (18.9)	2328 (19.8)	1901 (17.8)
Incident TB	Bacteriologically-confirmed	226 (1.0)	138 (1.2)	88 (0.8)
	Clinically diagnosed	114 (0.5)	50 (0.4)	64 (0.6)
	Unknown bacteriological status	71 (0.3)	49 (0.4)	22 (0.2)
	No incident TB diagnosed	22,013 (98.2)	11,498 (98.0)	10,515 (98.4)

SD: standard deviation; TB: tuberculosis; HIV: Human immunodeficiency virus.

Ascertainment of incident TB varied across studies (Table S1). Three studies identified incident TB through linkage with national or local TB surveillance databases or public health notification systems.^8^^,^^9^^,^^11^ Other studies used active outcome ascertainment, including periodic symptom assessment, scheduled study visits, direct contact with treating physicians, and microbiological evaluation for participants with suspected TB; three of them supplemented active follow-up with surveillance data or clinic TB register review.^7^^,^^12^^,^^15^ Two studies reported only bacteriologically confirmed incident TB,^12^^,^^13^ seven included both bacteriologically confirmed and clinically diagnosed TB,^8^, ^9^, ^10^^,^^14^, ^15^, ^16^, ^17^ and two did not have information on bacteriological confirmation status.^7^^,^^11^

Males comprised 48% (n = 10,689) of the study population. Baseline characteristics were largely similar between sexes. However, males were more likely to report ever smoking than females (n = 1875, 17.5% vs. n = 755, 6.4%).

Excluded participants had a higher proportion of people living with HIV, history of contact with a person with TB, ever smoking, previous TB diagnosis, and incident TB (Table S2). The excluded group also had a slightly higher proportion of females than the included group (n = 2922, 57.1% vs. n = 11,735, 52.3%).

### Prevalence of TB infection by sex

**Fig. 2:**
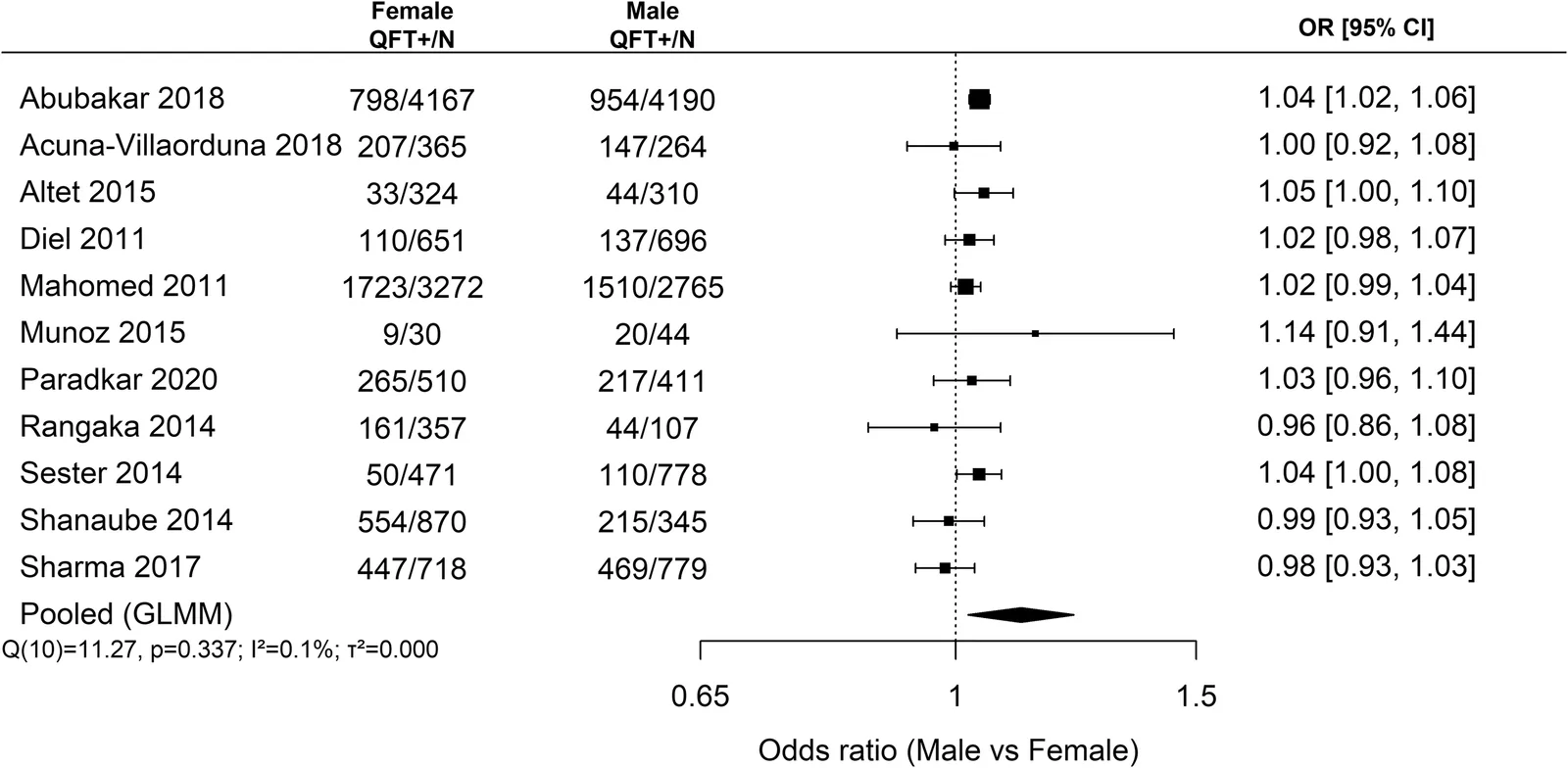
**Odds ratios for TB infection (male vs. female)**. Odds ratios were adjusted for age. TB: tuberculosis; OR: odds ratio; CI: confidence interval; GLMM: generalized linear mixed model.

The pooled prevalence of baseline TB infection across 11 studies was 35.0% (95% CI 22.8–49.4%) for female and 38.1% (95% CI 26.9–50.6%) for male (Figure S2). The likelihood of TB infection was significantly higher among males than females after adjustment for age, with a pooled odds ratio of 1.12 (95% CI 1.02–1.22) (Fig. 2).

### Risk of TB incidence by sex

**Fig. 3:**
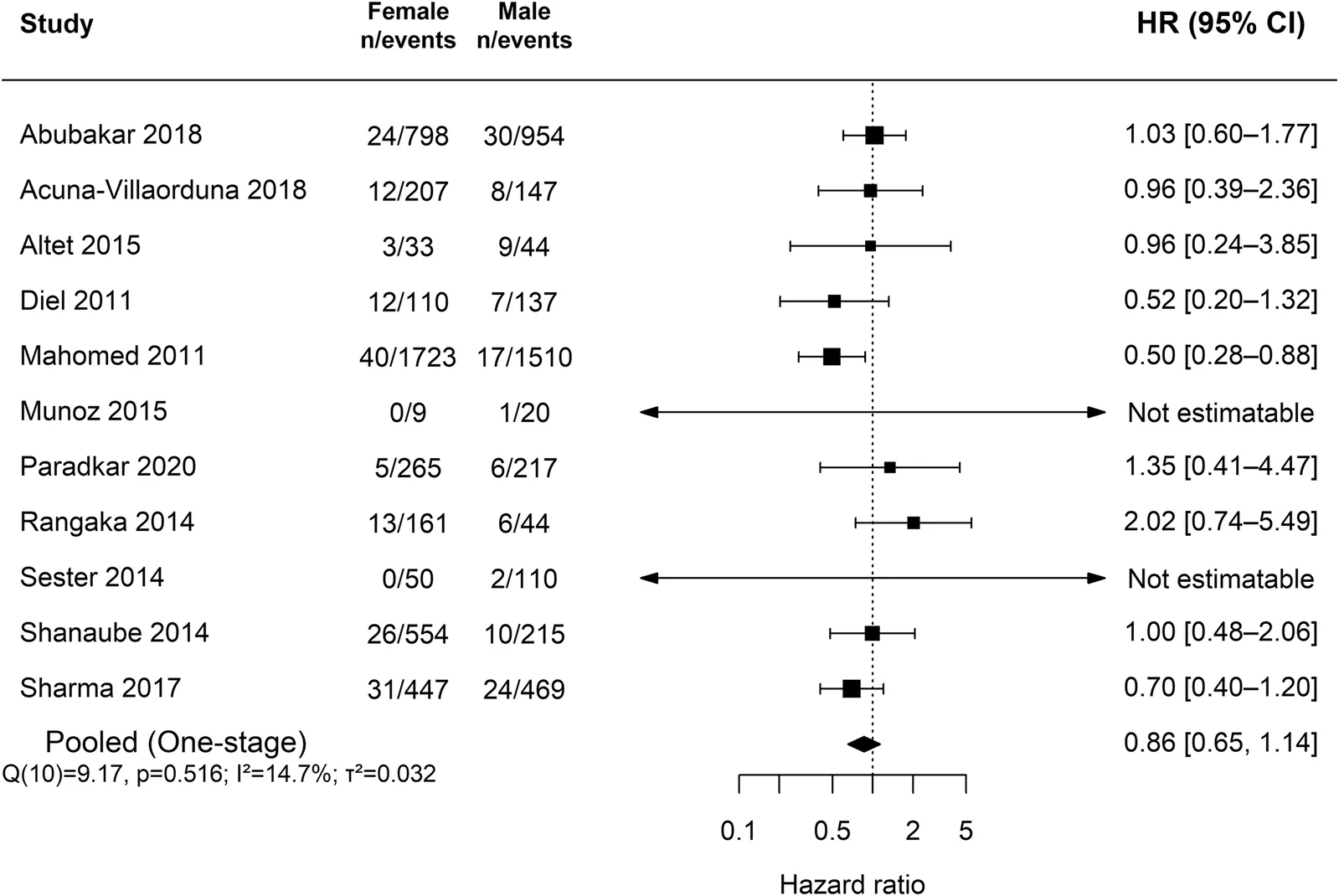
**Hazard ratios for incident TB (male vs. female) among participants with a positive TB infection test at baseline**. TB; tuberculosis: HR: hazard ratio; CI: confidence interval. Note: Hazard ratios were adjusted for age.

In participants with a positive infection test at baseline, TB incidence rates stratified by sex ranged from 3.6 to 61.8 cases per 1000 person-years (Figure S3). The pooled incidence rates were 16.3 cases per 1000 person-years (95% CI 10.9–24.4) among females and 15.6 cases per 1000 person-years (95% CI 9.8–24.8) among males. The pooled hazard ratio adjusted for age comparing males with females was 0.86 (95% CI 0.65–1.14) (Fig. 3). Study-specific hazard ratios were generally consistent with no substantial excess risk of TB among males. Point estimates ranged from 0.50 to 1.35 across most studies, with overlapping confidence intervals. One study among people living with HIV reported a higher point estimate (HR 2.02; 95% CI 0.74–5.49),^15^ although this estimate was imprecise. The I^2^ statistic was 14.7%. In two studies that included transplant recipients and/or other clinically immunocompromised groups in low TB incidence settings,^13^^,^^16^ study-specific hazard ratios could not be estimated because of sparse events, contributing limited information to the pooled estimate. There was no evidence of non-proportional hazards for sex (p = 0.59).

Among participants with a negative infection test at baseline, TB incidence rates were substantially lower than those observed among participants with a positive infection test, ranging from 0.3 to 36.3 cases per 1000 person-years (Figure S4). The pooled incidence rates were 2.6 cases per 1000 person-years (95% CI 0.8–8.8) among females and 3.5 cases per 1000 person-years (95% CI 1.4–8.6) among males. The pooled age-adjusted hazard ratio comparing males with females was 1.02 (95% CI 0.71–1.47). Study-specific hazard ratios ranged from 0.93 to 2.04, with wide confidence intervals, and several studies had non-estimable estimates due to small numbers or absence of TB events (Figure S5). There was no evidence of non-proportional hazards for sex (p = 0.70).

### Subgroup and sensitivity analyses

In subgroup analyses (Table S3), pooled hazard ratios comparing male to female TB incidence were generally consistent across most subgroups, including HIV-negative participants, adults, contacts and non-contacts, and studies stratified by national TB incidence, with estimates ranging from 0.80 to 0.96 and confidence intervals overlapping the null. In contrast, among people living with HIV, the pooled hazard ratio was higher (HR 1.91; 95% CI 0.97–3.74).

Data on bacteriologically confirmed incident TB were available from nine studies.^8^, ^9^, ^10^^,^^12^, ^13^, ^14^, ^15^, ^16^, ^17^ Among 341 incident TB cases in these cohorts, 226 (66.3%) were bacteriologically confirmed. When stratified by sex, 138 of 188 cases among females (73.4%) and 88 of 153 cases among males (57.5%) were bacteriologically confirmed. In a sensitivity analysis restricted to bacteriologically confirmed TB among participants with TB infection at baseline, males had a lower risk of developing TB compared with females (HR 0.64; 95% CI 0.46–0.89) (Table S3 and Figure S6). However, when the analysis was restricted to the same nine studies but included both bacteriologically confirmed and clinically diagnosed TB, the hazard ratio was closer to the primary analysis (HR 0.92; 95% CI 0.66–1.29).

In an additional sensitivity analysis using a two-stage meta-analysis and Poisson regression model, the estimates were similar to the primary analysis (Table S3).

## Discussion

In this pooled individual participant data analysis from 11 prospective cohort studies conducted across diverse geographical settings, no clear evidence of sex differences in the risk of developing TB was found after accounting for baseline TB infection status. Although males had a slightly higher prevalence of TB infection at baseline, the risk of progression to TB among individuals with baseline QFT positivity was similar between males and females. These findings suggest that the well-documented excess burden of TB among males appears more consistent with differences in TB infection risk than with a large sex difference in progression from infection to disease. However, baseline QFT status is an imperfect measure of infection, and we did not measure upstream determinants of infection risk, such as social mixing, occupational exposure, and other behavioural factors.

While we observed a higher prevalence of TB infection among adult males, our cohort primarily comprised high-risk groups, which may limit generalizability to the general population. However, higher male infection prevalence was also reported in a recent systematic review that included diverse populations and from national surveys.^4^ The review suggested that increased exposure and risk of infection are key determinants of sex disparities in TB notifications. Our results are concordant with this interpretation and further support the hypothesis that exposure-related factors—potentially including social mixing patterns, occupational exposures, and behavioural risk factors—contribute to higher infection risk among males.^23^ Recent studies of TB treatment outcomes also suggest that observed sex disparities may partly reflect social, behavioural, healthcare-related, and clinical factors. A recent multi-country study in Eastern Europe reported that while males often experience less favourable outcomes, this association was significantly attenuated after adjusting for socio-demographic and clinical factors.^24^ Similarly, a multicentre Italian cohort found that men were more likely to be lost to follow-up, whereas the risk of adverse events and microbiological response did not differ significantly by sex.^25^ These findings suggest that sex disparities in TB outcomes may not be explained by biological mechanisms alone.

The higher burden of TB among males is likely to reflect multiple mechanisms rather than a single pathway. Differences in exposure and acquisition of infection may contribute to higher baseline TB infection prevalence among males, while biological sex-related mechanisms may influence both the establishment of infection and progression from infection to disease. A recent review highlights potential roles for sex hormones, sex chromosomes, and sex-specific innate and adaptive immune responses in TB pathogenesis and outcomes, and notes that sex differences in TB burden become more pronounced after puberty.^26^ In our adult subgroup analysis, however, we did not observe clear evidence of a large excess risk of subsequent TB disease among males after accounting for baseline QFT status. Importantly, this finding does not exclude biological differences in progression risk, particularly given the limitations of the available cohorts. Rather, it suggests that, in these cohorts, the higher TB burden among males was more clearly reflected in higher baseline TB infection prevalence than in a large difference in subsequent TB disease risk after accounting for baseline QFT status.

There are limited data on incident TB risk by sex in well-characterized cohorts with explicit stratification by baseline infection status. An older study from the 1960s in India reported an approximately 3-fold higher risk of culture-positive TB in males over 15 years accounting for baseline TST results, which may reflect increased exposure during long-term follow-up.^27^ A more recent study in Brazil among people living with HIV reported higher TB incidence in males (IRR 1.33) despite similar baseline TST positivity (22.3% in males vs. 22.7% in females)^28^; however, females had higher mean CD4 counts (359 vs. 326 cells/μL). In our subgroup analysis of people living with HIV, males also showed a higher point estimate for TB risk (HR 1.91; 95% CI 0.97–3.74). However, this estimate was imprecise and should be interpreted cautiously. Our study utilized diverse populations from both high- and low-incidence settings and used QFT results to account for baseline infection. The absence of substantial sex differences in progression risk is consistent with earlier work. For example, in a TB risk prediction model developed using individual participant data from 18 cohorts—five of which overlap with the present analysis—sex was not retained in the final model due to its limited contribution to risk prediction.^29^ Similarly, in a prospective cohort study among household contacts in Peru, sex was not identified as a significant predictor of progression to TB.^30^

We observed no major differences in established TB risk factors between males and females, with the exception of smoking, which was more commonly reported among males. This likely reflects the composition of the included cohorts, which primarily comprised individuals from high-risk groups such as household contacts, migrants, and people with clinical risk factors for TB. Such selection may have resulted in a more homogeneous distribution of TB risk factors between sexes than would be observed in the general population, thereby attenuating potential sex differences in disease risk. Rather than representing a limitation, this finding highlights the challenges of disentangling inherent sex-related differences in TB risk using routine notification data, where differential exposure, infection risk, and the prevalence of comorbidities are difficult to adequately account for.

The similar risk of TB by sex was consistent across subgroups and under alternative model specifications. However, in the sensitivity analysis restricted to bacteriologically confirmed TB, males had a lower risk than females, a finding that was unexpected. When the analysis was restricted to the same nine studies but included both clinically diagnosed and bacteriologically confirmed TB, the estimate was closer to the primary analysis, suggesting that exclusion of studies alone did not explain this finding. The result may therefore reflect the more restrictive outcome definition, chance variation due to sparse events and multiple analyses, or differential bacteriological confirmation by sex. For example, males and females may have differed in adherence to, or completion of, diagnostic pathways after symptom onset. This finding should be interpreted cautiously and warrants further investigation in other cohort studies.

There are several limitations to this study. Despite the large overall sample size, TB events were relatively uncommon, particularly in analyses stratified by baseline TB infection status, HIV status, TB contact status, or restricted to bacteriologically confirmed TB. As a result, some subgroup and sensitivity analyses were based on sparse events, leading to increased statistical uncertainty. Second, we excluded participants who received TB preventive treatment. If initiation of preventive treatment differed by sex and was associated with underlying TB risk—for example, if males at higher risk of TB were more likely to receive preventive treatment—this exclusion could have introduced selection bias and influenced sex-specific estimates of TB incidence. However, the proportion of participants who received TB preventive treatment was small (<10%), and this is unlikely to have affected the results substantially. Third, not all included studies collected detailed or harmonised information on key covariates such as smoking intensity, alcohol use, socioeconomic status, or healthcare-seeking behaviour. This limited our ability to conduct more nuanced mediation analyses to separate biological effects of sex from gender-related pathways. Fourth, baseline QFT positivity does not indicate when infection occurred. Since progression risk is highest soon after infection,^31^ sex differences in recency of infection could have influenced the observed comparison of subsequent TB incidence among participants with baseline TB infection. We could not assess this directly in the available data. Fifth, three included studies relied solely on routine notification systems for outcome ascertainment. Because prevalence surveys suggest that males may have a larger burden of undiagnosed TB and greater gaps in detection or reporting than females,^2^ incident TB may have been differentially under-ascertained among males, potentially attenuating observed sex differences in TB incidence. Lastly, the original search was designed to identify cohorts evaluating the predictive performance of TST and IGRAs and was last conducted in September 2020. However, the original eligibility criteria were closely aligned with the data structure required for the present analysis, as studies had to include baseline TB infection testing and prospective follow-up for incident TB. Nevertheless, the included cohorts may not represent all populations in which sex differences in TB progression could be assessed. As discussed above, our cohort primarily comprised high-risk populations, such as contacts and migrants, in whom sex differences in TB exposure, risk factors, and disease risk may differ from those in the general population. The findings may therefore not be fully generalisable to the general population. To contextualise our findings, we conducted an updated MEDLINE search in January 2026 and identified two relevant studies that reported sex-specific TB risk while accounting for baseline infection status, one from India^27^ and one from Brazil,^28^ both of which are discussed above.

In conclusion, our pooled analysis of prospective cohort studies found no clear evidence of substantial sex differences in the risk of TB development, when stratified by baseline QFT status. However, we observed a higher prevalence of TB infection among males, consistent with findings from a previous systematic review. These findings suggest that differences in TB infection risk may be an important determinant of sex disparities in TB notifications globally, potentially reflecting differences in exposure risk. Together, these findings highlight the importance of addressing sex-specific patterns of TB exposure in prevention strategies.

## Contributors

YH and SS conceived the study with input from RKG, MQ, and MXR. YH and SS conducted the analyses with input from RKG and MQ. CAV, NA, RD, JD, SF, AG, ECJ-L, CL, FvL, ILR, VM, LMu, MP, MS, HP, KS, SKS, RS, GS, RV, IA, and MXR contributed data to the pooled analysis. YH and SS drafted the manuscript. MQ provided methodological input. YH and SS accessed and verified the data. All authors contributed to interpretation of the findings, critically reviewed the manuscript, and approved the final version for submission.

## Data sharing statement

The data used in this study are available upon reasonable request, subject to approval by the corresponding authors of the original studies.

## Declaration of interests

RKG reports support from NIHR (NIHR303184) for the present manuscript, and grants or contracts from the BMA Foundation for Medical Research, Wellcome Trust, and Royal Society outside the submitted work. RD reports honoraria from DiaSorin for a lecture. AG reports NIH grant funding paid to her institution. CL reports support from the German Center for Infection Research (DZIF) for the present manuscript; consulting fees from INSMED and bioMérieux; speaker honoraria from INSMED, Gilead, AstraZeneca, GSK, MedUpdate, and MedUpdateEurope; and participation in a WHO guideline development group, outside the scope of this work. VM, HP, and MP report NIH study grants paid to their institutions.

MS has received QuantiFERON kits for investigator initiated research projects carried out within the Tuberculosis network European Trialsgroup (TBnet) free of charge. Martina Sester has received grant support by Astellas, Biotest, and Takeda to the institution Saarland University outside of the submitted work, and has received honoraria for presentations or work in Data Safety monitoring Boards, and travel support for meetings by Biotest, Novartis, Takeda, MSD, and Moderna outside the submitted work.

The other authors have no competing interests to declare.
