# Supplementary material for: Sex differences in tuberculosis incidence: a pooled analysis of 11 prospective cohort studies
Source: eClinicalMedicine. 2026 Aug 12;99:104146. doi: 10.1016/j.eclinm.2026.104146 (PMC13616561; doi:10.1016/j.eclinm.2026.104146)
Supplement: Supplementary file 1 [file mmc1.docx]

**Appendix 1. Supplementary Figures and Tables**

**Figure S1. Conceptual framework illustrating hypothesised pathways linking sex to incident TB.**

*****Sex is conceptualised as encompassing both biological sex and socially patterned gender-related factors.

**Figure S2.Prevalence of TB infection by sex by study**

**Figure S3. TB incidence among participants with a positive TB infection test at baseline**

TB: tuberculosis; PY: person-year; IR: incidence rate; CI: confidence interval

**Figure S4. TB incidence among participants with a negative TB infection test at baseline**

TB: tuberculosis; PY: person-year; IR: incidence rate; CI: confidence interval

Note: Continuity corrections were applied to estimate incidence rates for individual studies with zero events; however, no continuity corrections were applied in the pooled analyses.

**Figure S5. Hazard ratios for incident TB by sex among participants with a negative TB infection test at baseline**

TB; tuberculosis: HR: hazard ratio; CI: confidence interval

Note: Hazard ratios were adjusted for age. In four studies, estimates could not be obtained because no TB events were observed in one or both sex strata.

**Figure S6. Hazard ratios for bacteriologically-confirmed incident TB by sex among participants with a positive TB infection test at baseline**

Note: Hazard ratios were adjusted for age.

**Table S1. Study characteristics**

| Study | N | Country | Study population | Outcome assessment | Outcome definition | Age,  mean (SD) | Female,  n (%) | HIV-positive,  n (%) | Receipt of TPT,  n (%) | Follow up,  median years (IQR) |
| --- | --- | --- | --- | --- | --- | --- | --- | --- | --- | --- |
| Abubakar 2018 | 9826 | UK | Contacts and migrants from high TB incidence countries | Linkage with the national TB database | BC or clinical TB | 37.0 (15.2) | 4898 (49.8) | 55 (0.6) | 259 (2.6) | 4.8 (3.9-5.5) |
| Acuna-Villaorduna 2018 | 890 | Brazil | Household contacts | Linkage with the TB surveillance database | BC or clinical TB | 26.1 (19.7) | 494 (55.5) | 0 (0.0) | 136 (15.3) | 5.2 (3.3-6.2) |
| Altet 2015 | 1258 | Spain | Close contacts | Periodic symptom assessment supplemented by surveillance database linkage | BC or clinical TB | 24.4 (15.3) | 623 (49.5) | 20 (1.6) | 623 (49.5) | 4 (4-4) |
| Diel 2011 | 1414 | Germany | Close contacts | Passive follow-up based on TB reports to the Public Health Department | Unknown BC status | 30.0 (11.8) | 684 (48.4) | No data | 67 (4.7) | 3.5 (2.5-4.2) |
| Mahomed 2011 | 6363 | South Africa | HIV-negative adolescents | Active or passive follow-up, supplemented by clinic TB record surveillance | BC TB only | 15.3 (1.5) | 3458 (54.3) | 1 (0.0) | 0 (0.0) | 2 (1.9-2) |
| Muñoz 2015 | 76 | Spain | Liver or stem cell transplant recipients | Medical chart review and contact with treating physicians | BC TB only | 54.3 (10.2) | 30 (39.5) | 0 (0.0) | 0 (0.0) | 4.3 (3.6-4.8) |
| Paradkar 2020 | 1009 | India | Household contacts | Periodic symptom assessment with microbiological evaluation for participants with suspected TB | BC or clinical TB | 27.3 (16.3) | 561 (55.6) | 15 (1.5) | 11 (1.1) | 1.9 (1.8-2) |
| Rangaka 2014 | 1324 | South Africa | People living with HIV in a randomized controlled trial | Three-monthly symptom assessment supplemented by national and provincial TB database surveillance | BC or clinical TB | 34.8 (8.3) | 996 (75.2) | 1324 (100.0) | 661 (49.9) | 2.6 (2.2-3.1) |
| Sester 2014 | 1462 | 11 European countries | Immunocompromised adults (people living with HIV, chronic renal failure, rheumatoid arthritis, and transplant recipients) | Incident TB actively ascertained through patient contact by treating physicians | BC or clinical TB | 49.6 (15.1) | 548 (37.5) | 712 (48.7) | 126 (8.6) | 2.8 (1.6-3.6) |
| Shanaube 2014 | 1350 | South Africa and Zambia | Household contacts (including HIV-positive and negative) | Follow-up visits at 18 and 36 months, supplemented by TB register review | Unknown BC status | 33.7 (15.6) | 966 (71.6) | 370 (27.4) | 0 (0.0) * | 2.4 (1.5-2.8) |
| Sharma 2017 | 1510 | India | Household contacts | Six-monthly active follow-up for TB symptoms | BC or clinical TB | 24.3 (15.2) | 726 (48.1) | 0 (0.0) | 0 (0.0) | 2 (2-2) |

*Missing data on the receipt of TB preventive treatment in 383/1350 (28.4%) participants.

HIV, human immunodeficiency virus; IQR, interquartile range; SD, standard deviation; BC, bacteriologically confirmed; TB, tuberculosis; TPT, tuberculosis preventive treatment

**Table S2. Characteristics included and excluded participants**

|  | | Level | Included | Excluded |
| --- | --- | --- | --- | --- |
| n | |  | 22424 | 5114 |
| Age (mean (SD)) | | | 29.69 (16.5) | 29.72 (16.6) |
| Sex | | Female | 11735 (52.3) | 2922 (57.1) |
|  | | Male | 10689 (47.7) | 2118 (41.4) |
|  | | Missing | 0 (0) | 74 (1.4) |
| HIV status (%) | | Negative | 18929 (84.4) | 3686 (72.1) |
|  | | Positive | 1491 (6.6) | 1260 (24.6) |
|  | | Missing | 2004 (8.9) | 168 (3.3) |
| History of contact with a person with TB (%) | | No | 10525 (46.9) | 1886 (36.9) |
|  | | Yes | 11891 (53.0) | 3226 (63.1) |
|  | | Missing | 8 (0.0) | 2 (0.0) |
| Smoking status (%) | | Ever smoker | 2630 (11.7) | 978 (19.1) |
|  | | Never smoker | 11280 (50.3) | 2704 (52.9) |
|  | | Missing | 8514 (38.0) | 1432 (28.0) |
| Body mass index (mean (SD)) | | | 24.08 (5.5) | 24.28 (5.8) |
|  | Missing (%) | | 7273 (32.4) | 2307 (45.1) |
| TB incidence rate (per 100K) in country of birth | | <100 | 5457 (24.3) | 1334 (26.1) |
|  | | 100-300 | 7770 (34.7) | 2262 (44.2) |
|  | | >300 | 8158 (36.4) | 1100 (21.5) |
|  | | Missing | 1039 (4.6) | 418 (8.2) |
| Reason for TB infection screening (%) | | Contact investigation | 10311 (46.0) | 3142 (61.4) |
|  | | Migrant screening | 4207 (18.8) | 326 (6.4) |
|  | | HIV | 1092 (4.9) | 950 (18.6) |
|  | | Immunosuppression (other than HIV) | 695 (3.1) | 132 (2.6) |
|  | | Research purpose (HIV-negative adolescents) | 6037 (26.9) | 544 (10.6) |
|  | | Missing | 82 (0.4) | 20 (0.4) |
| Previous TB diagnosis (%) | | No | 16964 (75.7) | 3476 (68.0) |
|  | | Yes | 1231 (5.5) | 482 (9.4) |
|  | | Missing | 4229 (18.9) | 1156 (22.6) |
| Study country (%) | | Brazil | 629 (2.8) | 305 (6.0) |
|  | | Europe | 11661 (52.0) | 2502 (48.9) |
|  | | India | 2418 (10.8) | 144 (2.8) |
|  | | South Africa or Zambia | 7716 (34.4) | 2163 (42.3) |
| Incident TB | | Bacteriologically-confirmed | 226 (1.0) | 121 (2.4) |
|  | | Clinically diagnosed | 114 (0.5) | 80 (1.6) |
|  | | Unknown bacteriological status | 71 (0.3) | 37 (0.7) |
|  | | No incident TB diagnosed | 22013 (98.2) | 4876 (95.3) |

**Table S3. Subgroup and sensitivity analyses: risk of incident TB by sex among participants with a positive TB infection test at baseline**

| **Subgroup** | **Studies, n** | **Participants, n** | **TB events, n** | **Hazard ratio (95% CI)** | **P value for interaction*** |
| --- | --- | --- | --- | --- | --- |
| HIV-negative | 10 | 7773 | 241 | 0.80 (0.59-1.08) | Not estimated |
| HIV-positive | 3 | 427 | 43 | 1.91 (0.97-3.74) |  |
| Contact | 8 | 4707 | 211 | 0.85 (0.62-1.18) | 0.90 |
| Non-Contact | 5 | 3517 | 75 | 0.87 (0.54-1.42) |  |
| TB incidence rate ≥ 100 per 100,000 population | 5 | 5605 | 178 | 0.80 (0.55-1.15) | 0.29 |
| TB incidence rate < 100 per 100,000 population | 6 | 2619 | 108 | 0.96 (0.66-1.41) |  |
| Adults | 11 | 4503 | 188 | 0.98 (0.73-1.32) | 0.94 |
| **Sensitivity analyses** | **Studies, n** | **Participants, n** | **TB events, n** | **Relative risk (95% CI)** |  |
| Bacteriologically confirmed TB | 9 | 7208 | 159 | HR 0.64 (0.46–0.89) |  |
| Two-stage meta-analysis | 11 | 8224 | 286 | HR 0.84 (0.64–1.10) |  |
| Poisson model | 11 | 8224 | 286 | IRR 0.83 (0.65-1.06) |  |

TB: tuberculosis; CI: confidence interval; HR: hazard ratio; IRR: incident rate ratio

**Note:** Subgroup analyses in children were not performed because of insufficient numbers.

*Interaction p values were estimated using within-study centred subgroup variables. Country-level TB incidence was defined at the study level, so its interaction was estimated as an exploratory between-study comparison. An interaction test was not performed for HIV status because only two studies included both HIV-positive and HIV-negative participants.
